# Cardiovascular magnetic resonance in women with cardiovascular disease: position statement from the Society for Cardiovascular Magnetic Resonance (SCMR)

**DOI:** 10.1186/s12968-021-00746-z

**Published:** 2021-05-10

**Authors:** Karen G. Ordovas, Lauren A. Baldassarre, Chiara Bucciarelli-Ducci, James Carr, Juliano Lara Fernandes, Vanessa M. Ferreira, Luba Frank, Sophie Mavrogeni, Ntobeko Ntusi, Ellen Ostenfeld, Purvi Parwani, Alessia Pepe, Subha V. Raman, Hajime Sakuma, Jeanette Schulz-Menger, Lilia M. Sierra-Galan, Anne Marie Valente, Monvadi B. Srichai

**Affiliations:** 1 University of Washington, Seattle, USA; 2 Yale University, New Haven, CT USA; 3 Bristol Heart Institute, Bristol, UK; 4 Bristol National Institute of Health Research (NIHR) Biomedical , Research Centre, Bristol, UK; 5 University Hospitals Bristol, Bristol, UK; 6 University of Bristol, Bristol, UK; 7 Department of Radiology, Northwestern University Feinberg School of Medicine, Chicago, IL USA; 8 Jose Michel Kalaf Research Institute, Radiologia Clinica de Campinas, São Paulo, Brazil; 9 Oxford Centre for Clinical Magnetic Resonance Research (OCMR), Division of Cardiovascular Medicine, British Heart Foundation Centre of Research Excellence, Oxford NIHR Biomedical Research Centre, University of Oxford, Oxford, UK; 10 Medical College of Wisconsin, Wisconsin, USA; 11 Onassis Cardiac Surgery Center, Athens, Greece; 12 Kapodistrian University of Athens, Athens, Greece; 13 University of Cape Town, Cape Town, South Africa; 14 Groote Schuur Hospital, Cape Town, South Africa; 15 Department of Clinical Sciences Lund, Clinical Physiology, Skåne University Hospital Lund, Lund University, Lund, Sweden; 16 Division of Cardiology, Department of Medicine, Loma Linda University Health, Loma Linda, CA USA; 17 Magnetic Resonance Imaging Unit, Fondazione G. Monasterio C.N.R., Pisa, Italy; 18 Krannert Institute of Cardiology, Indiana University, Indianapolis, USA; 19 Department of Radiology, Mie University School of Medicine, Mie, Japan; 20 harite Hospital, University of Berlin, Berlin, Germany; 21 HELIOS-Clinics Berlin-Buch, Berlin, Germany; 22 American British Cowdray Medical Center, Mexico City, Mexico; 23 Boston Children’s Hospital, Brigham and Women’s Hospital, Boston, USA; 24 Georgetown University School of Medicine, Washington, USA

**Keywords:** Cardiovascular magnetic resonance, Women, Cardiovascular disease, Society for Cardiovascular Magnetic Resonance, Position statement

## Abstract

This document is a position statement from the Society for Cardiovascular Magnetic Resonance (SCMR) on recommendations for clinical utilization of cardiovascular magnetic resonance (CMR) in women with cardiovascular disease. The document was prepared by the SCMR Consensus Group on CMR Imaging for Female Patients with Cardiovascular Disease and endorsed by the SCMR Publications Committee and SCMR Executive Committee. The goals of this document are to (1) guide the informed selection of cardiovascular imaging methods, (2) inform clinical decision-making, (3) educate stakeholders on the advantages of CMR in specific clinical scenarios, and (4) empower patients with clinical evidence to participate in their clinical care. The statements of clinical utility presented in the current document pertain to the following clinical scenarios: acute coronary syndrome, stable ischemic heart disease, peripartum cardiomyopathy, cancer therapy-related cardiac dysfunction, aortic syndrome and congenital heart disease in pregnancy, bicuspid aortic valve and aortopathies, systemic rheumatic diseases and collagen vascular disorders, and cardiomyopathy-causing mutations. The authors cite published evidence when available and provide expert consensus otherwise. Most of the evidence available pertains to translational studies involving subjects of both sexes. However, the authors have prioritized review of data obtained from female patients, and direct comparison of CMR between women and men. This position statement does not consider CMR accessibility or availability of local expertise, but instead highlights the optimal utilization of CMR in women with known or suspected cardiovascular disease. Finally, the ultimate goal of this position statement is to improve the health of female patients with cardiovascular disease by providing specific recommendations on the use of CMR.

## Introduction

Cardiovascular magnetic resonance (CMR) imaging is a robust, reproducible and safe method for non-invasive evaluation of cardiovascular diseases (CVD). Strengths of CMR include precise assessment of cardiac anatomy and function [1] and multi-parametric tissue characterization [2]. CMR is recommended for diagnosis and management of multiple CVDs [3–5]. CMR is particularly suitable for the evaluation of women with suspected or known CVDs given the lack of ionizing radiation exposure, which can be potentially harmful to female breast tissue [6, 7] or to the fetus of pregnant patients [8]. This is an advantage of CMR compared to radiation-based imaging methods including nuclear medicine scintigraphy such as single-photon emission computerized tomography (SPECT), positron emission tomography (PET), coronary computed tomography angiography (CCTA) and catheter-based X-ray angiography.

Recent advances in tissue characterization and ischemia assessment make CMR an attractive tool for imaging various pathological aspects of cardiovascular disease in women [9–13].

## Aims and structure of the document

This document provides recommendations for clinical utilization of CMR in women with known or suspected CVD. We cite published evidence when available and provide expert consensus where there is paucity of evidence. Most of the evidence available pertains to translational studies involving subjects of both sexes. However, we have prioritized review of data obtained from female patients, and particularly valued the evidence providing direct comparison of CMR between women and men.

The women in CMR section of the SCMR has led various educational and advocacy initiatives to promote improved cardiovascular care of females with cardiovascular diseases. The group has recently published a comprehensive review paper highlighting the advantages of CMR for imaging cardiovascular disease in women [14]. Building on this initiative, the SCMR Consensus Group on CMR Imaging for Female Patients with Cardiovascular Disease was formed to provide guidance for current clinical CMR utilization for this purpose. Our committee is primarily composed of experts with a wide and representative range of training and expertise, a broad geographical representation and a balanced spectrum of clinicians and scientists.

The goals of this document, specific for the use of CMR in women with known or suspected CVD, are to (1) guide the informed selection of cardiovascular imaging methods, (2) inform clinical decision-making, (3) educate stakeholders on the advantages of CMR in specific clinical scenarios, and (4) empower patients with clinical evidence to participate in their clinical care.

### Statement of clinical utility

1. CMR can be used to diagnose ischemic myocardial injury in women with suspected acute coronary syndrome (ACS), which is particularly important, as they are more likely to present with non-obstructive coronary arteries compared to men. In addition, CMR can reliably establish an alternate diagnosis in women with ACS symptoms and suspected diagnosis of myocardial infarction with non-obstructive coronary arteries (MINOCA), such as myocarditis and Takotsubo cardiomyopathy, which can guide subsequent management and may impact their prognosis. (Gender neutral evidence).
2. CMR is recommended for diagnosing stable ischemic heart disease (IHD) in women. Compared to other non-invasive imaging techniques, CMR provides high accuracy with no sex differences in performance for diagnosis and prognosis in identifying both macrovascular and microvascular coronary artery disease (CAD). (Gender neutral evidence)
3. CMR is the recommended method to confirm the diagnosis and severity of peripartum cardiomyopathy (PPCM). Detection of presence and extent of myocardial damage is valuable for prediction of cardiac dysfunction and risk stratification in current and future pregnancies. (Gender specific evidence)
4. CMR can be used to diagnose cancer therapy-related cardiac dysfunction (CTRCD), especially in breast cancer patients, and to confirm echocardiography-diagnosed left ventricular (LV) dysfunction. CMR is recommended for myocardial tissue characterization in the diagnosis and treatment workup to assess the etiology of cardiomyopathy. (Gender neutral evidence)
5. CMR including CMR angiography (CMRA) is the preferred imaging modality for serial concomitant assessment of aortic valve function and thoracic aorta dimensions in patients with bicuspid aortic valve (BAV) and aortopathies. (Gender neutral evidence)
6. In women who are planning to become or are currently pregnant, CMR is recommended in those with aortic syndrome, known aortic disease and congenital heart disease (CHD) when echocardiography is suboptimal. CMR provides assessment of the entire aorta, precise quantification of ventricular function, and localization of left ventricular outflow tract (LVOT) obstruction to inform decision making during pregnancy. (Gender specific evidence)
7. CMR is indicated for combined functional evaluation and tissue characterization of the myocardium and vasculature in patients with systemic rheumatic diseases and collagen vascular disorders, as CMR can detect associated myocardial inflammation, provides important prognostic information and may lead to changes in both cardiac and systemic treatment strategies. (Expert opinion)
8. CMR is a useful and often an essential modality for early detection of myocardial disease in female carriers of cardiomyopathy-causing mutations and could guide deployment of effective therapies. (Expert opinion)

## Imaging females with acute coronary syndrome

### Clinical scenario

ACS is a leading cause of death worldwide [15]. ACS comprise of clinical presentation consistent with acute ischemia and includes ST-segment elevation myocardial infarction (STEMI), Non- ST-segment elevation myocardial infarction (NSTEMI) and unstable angina.

*Sex differences/prevalence* There are recognized sex differences in the presentation, diagnosis, management and treatment of ACS and myocardial infarction (MI) [16–18]. Women are less likely to present with chest pain and often have atypical symptoms, leading to underdiagnosis, especially in younger women [19, 20]. Women who do present to the emergency department with chest pain are investigated less aggressively than men, are less likely to receive a correct diagnosis, and are not referred as often for guideline-recommended diagnostic and/or therapeutic procedures [21]. Women with ACS are not as likely to have significant obstructive CAD on invasive coronary angiography and more likely to have ruptured plaque, plaque erosion, or thrombus formation [22, 23]. Among women with MI and MINOCA, a significant proportion show biochemical or imaging evidence of myocardial ischemia [22]. Spontaneous coronary artery dissection [24], coronary thromboembolism, coronary artery vasospasm, microvascular dysfunction, and Takotsubo stress-induced cardiomyopathy, are a few of the ACS etiologies that need special consideration in women.

*Traditional evaluation* The standard work-up includes assessment of risk factors, history and physical examination, blood biomarkers for cardiomyocyte damage (e.g., troponins) and the electrocardiogram (ECG), Invasive coronary angiography, non-invasive cardiac imaging modalities such as echocardiography, CCTA and CMR. Advanced coronary imaging techniques such as optical coherence tomography (OCT), intravascular ultrasound (IVUS) are the cornerstone of diagnosis in cases with ACS with no or < 50% obstructive CAD. These tests may help confirm, refine or reclassify the initial diagnosis, which determines treatment pathways and outcomes.

*Benefits for use of CMR* *(gender neutral evidence)* CMR provides significant incremental value to conventional tests in establishing an accurate diagnosis [25–27] and prognosis in ACS [28]. CMR plays an important role in the contemporary assessment and prognosis of MINOCA [26, 29–31]. CMR allows detection of small MIs, distinguishing between left anterior descending coronary artery (LAD) territory infarction and Takotsubo cardiomyopathy, and differentiating MI from myocarditis. Since women are more likely to present in an atypical manner, they should be approached differently than men. CMR may help to confirm (or exclude) a diagnosis of ACS, so that physicians may provide appropriate timely treatment.

### Suggested protocol

- Balanced steady-state free precession (bSSFP) cine (short and long axis planes) for evaluation of cardiac structure, function and potential mechanical complications from acute MI
- T2-weighted (T2w) imaging for edema imaging according to current expert recommendations [30]
- T2* mapping for detection of intramyocardial hemorrhage[32] (optional)
- T1-weighted (T1w) late gadolinium enhancement (LGE) is the recommended methodology to estimate the extent of acute myocardial necrosis and microvascular obstruction (MVO) [32]
- T1w early gadolinium enhancement (EGE) may detect LV intracavitary thrombus (can also visualize MVO) (optional)
- In stable patients outside of the acute phase, consideration should be given to vasodilatory stress perfusion imaging (adenosine, regadenoson, dipyridamole) with gadolinium based contrast agent (GBCA) to assess for the functional significance of any bystander CAD, and, in particular, microvascular dysfunction.
- Parametric mapping techniques (T1/T2/ extracellular volume fraction (ECV)) are emerging CMR technologies that hold promise in providing more sensitive detection of acute myocardial injury, including assessment of the extracellular volume and myocardial interstitium that may provide further insights into differences between women and men who present with ACS.

### Stable ischemic heart disease—imaging ischemia

*Clinical scenario:* IHD is the leading cause of cardiovascular death worldwide [33]. Stable IHD includes patients who have had ACS or are at risk for ACS, even if they have no or limited symptoms. In 2015, there was an estimated 110 million patients with IHD worldwide. IHD accounts for the majority of CVD in patients over 40 years, with steady increase in prevalence with age [34]. Early detection of these patients is important to decrease morbidity and mortality [35]

*Sex differences/prevalence:* Women have slightly lower prevalence of IHD, but higher mortality rate and complications, indicating a potential gap in accurate diagnosis and management strategies specific to women [15]. In comparison to men, female specific features include a higher proportion of symptomatic patients with non-obstructive CAD and microvasculature dysfunction [36], and more frequent presentation with “atypical” angina symptoms [37]. As a result, women tend to receive less intensive care that lead to higher morbidity, including heart failure, and mortality compared to men [38, 39].Given the higher dissociation between symptoms, ischemia burden and obstructive versus non-obstructive CAD in women, a more sensitive and “specific” functional evaluation of IHD is warranted for tailored therapeutic decisions [40].

*Traditional evaluation:* Diagnostic testing is recommended in symptomatic stable patients with suspected or known IHD and change in clinical status. Functional assessment for ischemia with stress testing with or without imaging is the most common method of evaluation. The advent of CCTA allows for direct, noninvasive evaluation of the underlying anatomic coronary features in IHD and provides an alternative diagnostic method [35].

*Benefits for use of CMR* *(gender neutral evidence):* Poorly calibrated pre-test probability tables in addition to limitations with achievement of maximum exercise capacity are factors that lead to the relatively low diagnostic yield of traditional tests like exercise treadmill stress test and stress echocardiography for females with IHD [41, 42]. CCTA provides high anatomic correlation with invasive X-ray coronary angiography, but given the higher dissociation between symptoms, ischemia burden and obstructive versus non-obstructive CAD in women, functional evaluation of IHD is still warranted for proper therapeutic management decisions (Class I, level of evidence B) [43, 44], especially for women. CMR has many advantages over other modalities including the lack of ionizing radiation, greater sensitivity independent of the prevalence of the disease compared to SPECT with no sex differences in diagnostic performance [45], ability to identify microvascular dysfunction and provide automated quantitative evaluation of global and regional perfusion [46, 47], identification of concurrent myocardial scar [9, 48–50], and comprehensive evaluation to exclude other cardiac (myocardium, pericardium, valves or great vessels) causes of chest pain. While exercise stress CMR can be performed with suitable accuracy [51], more common approaches involve vasodilator stress CMR using adenosine, regadenoson or dipyridamole [41]. In the latest version of the 2004 American College of Cardiology/American heart Association (ACC/AHA) guidelines for stable CAD, pharmacological stress CMR is recommended for: diagnosis of IHD in patients with either known or intermediate to high pretest probability of IHD who are unable to exercise or have uninterpretable ECG (Class IIa, level of evidence B); risk assessment in patients with known CAD being considered for revascularization with unclear physiologic stenosis (Class I, level of evidence B); and follow-up of revascularized stable CAD in symptomatic patients (Class I, level of evidence C) [35, 42]. In the 2019 guidelines from the European Society of Cardiology (ESC) of chronic CAD, CMR is recommended for evaluation of patients with suspected coronary microvascular angina (Class IIb, level of evidence B) [44].

### Suggested protocol

- Rest bSSFP cine (short and long axis planes) for evaluation of cardiac structure and function
- Vasodilator stress using conventional doses and protocols: induced vasodilation (adenosine, regadenoson, dipyridamole) [52]
- Stress first pass perfusion imaging; automated quantitative perfusion mapping is recommended if available
- T1w LGE (short and long axis planes)
- Native T1-mapping and stress T1-mapping are emerging technologies that may play a role in the GBCA-free CMR assessment of ischemic heart disease [53, 54]. ECV may serve as surrogate marker for diffuse fibrosis, although resting coronary vasodilation associated with ischemia may expand the intravascular compartment (thus ECV) in the ischemic territory.

### Peripartum cardiomyopathy

*Clinical scenario:* Peripartum cardiomyopathy (PPCM) is a rare form of dilated cardiomyopathy characterized by LV systolic dysfunction and LV ejection fraction (LVEF) < 45% occurring during late pregnancy or in the months following delivery in previously healthy women without a history of heart disease. Right ventricular (RV) systolic dysfunction may be noted in up to 35% of PPCM patients [55] and is linked to worse prognosis than isolated LV involvement. Symptoms are non-specific, such as shortness of breath, cough, orthopnea, and hemoptysis. Patients may also present with arrhythmias, embolic events, and myocardial infarction.

Multiple theories exist to explain PPCM, including genetic predisposition, apoptosis, and abnormal immune response to fetal microchimerism, abnormal hemodynamic response, prolonged tocolysis, and ethnicity, among others [56]. Risk factors predisposing for PPCM are advanced age, multiparity and twin pregnancies, African American ethnicity, hypertension, and preeclampsia [57]

*Prevalence:* PPCM is a female specific disease with mixed and inconsistent reports of incidence. Two of the largest population-based studies by Mielniczuk, [58] and Brar [59] report mortality of 2.05% in a retrospective study of 3.6 million patients, with an incidence of approximately 1 per 3189 live births [55] and mortality of 3.3% in 241,497 patients with an incidence of 1 in 4025 live births. Incidence greatly varies by ethnicity, with lowest reported among Hispanic women and highest among African American women.

*Traditional evaluation:* PPCM is diagnosed during current or recent pregnancy and symptoms of heart failure in previously healthy woman, family history of heart disease, abnormal ECG, elevated troponins, and N-terminal pro-B-type natriuretic peptide (NT-proNBP). Imaging assessment of PPCM is performed using echocardiography as the primary modality, CMR for confirmation of diagnosis and assessment of severity, and rarely cardiac catheterization/endomyocardial biopsies. Therapeutic interventions consider wellbeing of both mother and the fetus by informing best type and dosage of medications. Some patients require continuous monitoring until improvement of cardiac function is achieved.

*Benefits for use of CMR in this scenario* *(gender specific evidence):* Assessment of heart disease without ionizing radiation exposure make CMR an optimal imaging modality in pregnancy. Furthermore, CMR helps evaluate the other etiology for dilated cardiomyopathy. Comprehensive assessment of cardiac function and myocardial damage by CMR assists planning and follow-up management of PPCM along with prediction of possible PPCM in subsequent pregnancies. The pattern of LGE in PPCM is nonspecific and often similar to idiopathic nonischemic cardiomyopathy. While some studies failed to demonstrate presence of LGE in peripartum cardiomyopathy [60], others report LGE in up to 40% of cases [61].When present, pattern of LGE shows focal and linear distribution, involving mid myocardial wall and sub-epicardium [60]^61^. The presence of LGE is associated with increased risk of heart failure decompensation during delivery, increased rate of readmissions, and increased risk for exacerbation during recurrent pregnancies [62]. Inflammatory reaction should be assessed to characterize reversible injury.

### Suggested protocol

- bSSFP cine (short and long axis planes) for evaluation of cardiac structure and function
- Detection of myocardial inflammation/edema using a T2-based method (e.g., T2w imaging or T2-mapping)
- Detection of myocardial edema, hyperemia/capillary leak, necrosis and fibrosis using a T1-based method (e.g., EGE, T1-mapping, ECV and/or T1w LGE) according to current expert recommendations [63]

### Assessment of cancer therapy-related cardiac dysfunction (CTRCD) in breast cancer patients

*Clinical scenario:* Chemotherapy, radiation therapy, and targeted agents have led to significant improvements in the survival rate of women with breast cancer [64]. However, these therapies are associated with on-target and off-target cardiac side effects, leading to CTRCD and substantial cardiac morbidity and mortality. 5-year cumulative incidence of heart failure and cardiomyopathy in women receiving combination therapy ranged from 7.5% in the young to 36% in the older patients [65]. In the latter, CVD is the leading cause of death (15.9%), with breast cancer being close but second (15.1%) [66].

*Prevalence:* Breast cancer is a female predominant disease, affecting about 1 in 8 women over the course of their lifetime, whereas the risk is 1 in 1000 for men [64]. There are reported sex disparities in the incidence of cancers in other organs, with generally higher incidence in men for most malignancies except for thyroid cancer which is more prevalent in women [67].

*Traditional evaluation:* Guideline driven evaluation and monitoring of cardiac function of women undergoing oncologic treatment is traditionally performed with transthoracic echocardiography (TTE), and preferably with three-dimensional (3D) LVEF and LV strain assessment [68–70]. A 10% change in LVEF may dictate the therapeutic regimen [70]. However, up to 25% of patients with cardiac dysfunction (LVEF < 50%) are missed with two-dimensional (2D) TTE [71]. In addition, 2D echocardiography has higher temporal variability (9.8%) than 3D echocardiography (5.6%) [72].which should be kept in mind when evaluating these patients. LVEF can equally be assessed accurately with multi-acquisition gated angiography (MUGA) as well as CMR and 3D echocardiography [73]. However, MUGA is not preferred owing to the ionizing radiation, which is a significant concern in women with breast cancer.

*Benefits for use of CMR in this scenario* *(gender neutral evidence):* CMR can be used as a first line method or when echocardiographic image quality is inadequate for volumetric and functional assessment of patients at risk for CTRCD, according to societal guidelines [74]. Furthermore, when echocardiography is abnormal (e.g., change in LVEF), CMR should be performed to confirm the altered LVEF prior to making decisions about management. Additionally, CMR should be used for tissue characterization and evaluation of ischemic and non-ischemic scar or fibrosis and inflammation, such as may occur in trastuzumab related cardiomyopathy and immune-checkpoint inhibitor myocarditis [75]. CMR strain imaging has been shown to decrease post anthracycline use [76] although currently no inter-vendor normal reference values has been established to guide management. T2w imaging with increased signal intensity have been observed in patients post anthracycline and trastuzumab [77, 78]. T1 mapping and ECV has been observed to increase after anthracycline exposure [79, 80], although their clinical significance and influence on patient management is yet to be determined. Stress perfusion CMR to detect or exclude functionally significant CAD in women [45] should be added if there is suspicion for or if necessary to exclude CAD prior to proceeding with surgery or other therapy where indicated.

### Suggested protocol

- Non-contrast study for those receiving serial imaging for screening of LV dysfunction in lieu of echocardiography
- bSSFP cine (short and long axis planes) for evaluation of cardiac structure and function
- Consider further tissue characterization for myocardial inflammation and other changes using T2w imaging and/or parametric T1/T2 mapping and ECV (optional)
- T1w LGE in suspected cardiomyopathy
- Stress first-pass perfusion to assess for functionally significant CAD prior to surgery or other therapy if indicated

### Baseline and follow aortic assessment in bicuspid aortic valve

*Clinical scenario:* Bicuspid aortic valve (BAV) disease affects 0.5–1% of the population with a male predominance of 3:1 [81]. BAV is associated with aortic valve dysfunction (i.e. stenosis or regurgitation) and thoracic aortic disease, including ascending aortic aneurysm and aortic dissection [82]. Thoracic aortic dilatation is thought to be independent of degree of valve stenosis and has been associated with reduced fibrillin in the aortic wall and/or genetic defects (e.g. NOTCH 1 gene) [83]. Many women with BAV are asymptomatic, however, when they do develop symptoms, they are related to aortic valve dysfunction, aortopathy (i.e., aneurysm, dissection) and acquired conditions such as endocarditis. Guidelines recommend screening first-degree relatives of patients with BAV for BAV and associated aortopathy [84]. Patients with BAV should be monitored for progressive aortic valve dysfunction and aortic disease. Surveillance is commonly performed with serial imaging at intervals between 6 months and 5 years depending on patient age and degree of underlying pathology [84, 85].

*Sex differences/prevalence:* Morphology type of BAV is similar between men and women. At initial presentation, men more frequently demonstrate moderate to severe aortic regurgitation, whereas women more commonly show moderate to severe aortic stenosis. Women are less likely to develop endocarditis or concomitant aortopathy compared to men [86, 87]. Despite less baseline morbidity compared to men, women exhibit a significantly higher mortality in tertiary and surgical referral cohorts, which can be independently predicted by presence of significant aortic regurgitation and/or New York Heart Association (NYHA) Class 3 or 4 heart failure symptoms [87]. The reasons for the excess mortality in women may relate to use of absolute as opposed to indexed cutoff values for referral to aortic valve or aorta surgery in which men will reach earlier than women as they have larger body surface area (BSA) values. Pregnancy in women with BAV is associated with an increased risk for complications [88] and women who may become or are pregnant should receive appropriate counseling (see section on “Pregnancy risk stratification for congenital heart disease and aortopathy”).

*Traditional evaluation:* Echocardiography is the preferred method for diagnosing BAV disease with reported high sensitivity, although diagnosis can be limited in patients with heavily calcified valves [89]. Visualization of the thoracic aorta is limited to the aortic root and ascending aorta with TTE. If the ascending aorta is not well visualized on echocardiography, then computed tomography angiography (CTA) or magnetic resonance angiography (MRA) is recommended. ECG gated CTA provides excellent visualization of the entire thoracic aorta with high resolution but has the disadvantage of radiation exposure and requirement for iodinated contrast. Assessment of the aortic valve with CTA requires a retrospectively gated acquisition, generally associated with higher radiation exposure, which is particularly relevant in young women exposure of the breast tissue (see section on “Radiation”). For patients with BAV, annual CTA or MRA is recommended if aortic root or ascending aorta is 3.5–4.4 cm, and with echocardiography to follow valve disease, if needed; for dimensions of 4.5–5 cm, bi-annual CTA or MRA is recommended.

*Benefits for use of CMR* *(gender neutral evidence):* CMR and MRA have the advantage of providing a comprehensive assessment of the aortic valve and the entire thoracic aorta in a single examination without radiation exposure. Direct planimetric measurement of the aortic valve area can be achieved using bi-orthogonal short axis cine images of the aortic valve. Non-contrast MRA techniques are available which mitigates any risk related to gadolinium deposition, which may be of particular concern with serial surveillance.

### Suggested protocol

- bSSFP cine (short and long axis planes) for evaluation of cardiac structure and function. Multiplanar bSSFP cine with specific views of the aortic valve and aorta
- Axial T1w fast spin echo through aorta (optional, for intramural hematoma, dissection)
- Axial T2w gradient echo through aorta (optional, for aortitis)
- 2D phase contrast velocity encoding at aortic root for aortic flow (or four-dimensional flow if available)
- 3D contrast enhanced or non-contrast MRA of the thoracic aorta
- Orthogonal dimensions of the aorta should be obtained from 7 anatomic landmarks in the thoracic aorta, according to the American Heart Association guidelines [90]

### Pregnancy risk stratification for congenital heart disease and aortopathy

*Clinical scenario:* CVD is a leading cause of morbidity and mortality in pregnant women [91]. Women with CVD particularly women with congenital heart disease (CHD) who are contemplating pregnancy should undergo a complete work-up including appropriate cardiac imaging for risk stratification prior to conception. For women with connective tissue disorders, pregnancy may be associated with progressive dilatation of the aorta secondary to estrogen effects, increased protease activity in the extracellular matrix and defective collagen synthesis [92]. Aortic dissection remains the greatest concern as it is associated with high maternal mortality, with increased risk in both the antepartum and postpartum periods [93]. Syndromes with high risk for aortic dissection include Marfan [94], Loeys-Dietz and Ehler Danlos type IV (vascular type). Other syndromes in which there is an increased risk of dissection include BAV and Turner syndrome. In women with Marfan syndrome, the aortic root size may not return to the pre-pregnancy dimensions and there appears to be an increased risk of future aortic events or need for aortic root replacement after pregnancy [95].

*Prevalence:* Approximately 1–4% of pregnant women have underlying CVD [96]. The rising incidence of CVD in pregnancy is related to a number of factors including advancing maternal age with associated increased prevalence of CVD risk factors and increasing number of women with CHD who reach childbearing age. CHD comprises at least 50% of CVD in pregnancy [97].

*Traditional evaluation:* Imaging is an important component of the evaluation before and during pregnancy [98]. Several risk stratification models have been developed summarizing maternal and fetal outcomes in women with CVD [99]^100^. Specific imaging factors included in these models that confer a higher risk of adverse events in pregnancy include depressed systemic ventricular function, obstructive morphology, pulmonary hypertension and aortopathy [100, 101].While TTE remains the first line imaging modality for CVD in pregnancy, many women with CHD undergo CMR for cardiovascular surveillance and risk stratification prior to pregnancy [98]

*Benefits for use of CMR (gender specific evidence): *In specific CHD populations, CMR studies have identified risk factors for adverse maternal outcomes in pregnancy. As an example, descending thoracic aortic diameter < 1.2 cm was correlated with higher maternal cardiovascular events in patients with aortic coarctation [99] and maternal cardiac events (cardiac arrest and arrhythmias) were associated with a systemic RV ejection fraction (RVEF) of < 35% [99] in women with atrial switch procedure for transposition of great arteries. CMR is particularly useful in women with aortic disease during pregnancy. The 2018 ESC Guidelines for Management of Cardiovascular Disease during Pregnancy states that imaging of the entire aorta with CMR or CTA is recommended before pregnancy in patients with a genetically proven aortic syndrome or known aortic disease (Class I, level of evidence C). During pregnancy, CMR (without GBCA) is recommended for imaging the distal ascending aorta, aortic arch, or descending aorta (Class I, level of evidence C) or when other non-invasive diagnostic testing is not sufficient for definitive diagnosis (Class IIa, level of evidence C) [96, 102]. The use of GBCA should be avoided, if possible, especially in the first trimester. A recent multicenter study including 83 pregnant women with vascular disease, CHD and cardiomyopathies has shown that management plans were changed in 35% of patients based on the CMR findings [103].

### Suggested protocol

- Patients should be imaged supine position.
- For patients > 20 weeks’ gestation, the use of a wedge or pillow under right buttock to tilt pelvis off the vena cava is recommended.
- If GBCA is necessary to answer the clinical question, a low-risk agent with the minimum possible amount should be used.
- 3D MRA of the chest can be performed with GBCA or using inherent increased signal of the blood pool with navigator-compensated and ECG-gated 3D bSSFP sequence.
- bSSFP cine sequences in the axial, oblique sagittal, and aortic orifice view planes are useful for precise measurement of maximum aortic size, particularly at the sinus of Valsalva where the most motion artifact is seen with standard 3D MRA.
- bSSFP cine (short and long axis planes) for evaluation of cardiac structure and function
- If there are concerns about the patient, communication with the obstetrical team is critical.
- Parametric T1 and T2 mapping holds promise to provide GBCA-free myocardial tissue characterization in the future, and may be particularly suitable for this patient group.

### Cardiovascular assessment in patients with systemic diseases

*Clinical scenario:* Rheumatoid arthritis, spondyloarthropathies, systemic lupus erythematosus, systemic vasculitis, inflammatory myopathies, systemic sclerosis and mixed connective tissue disease are autoimmune rheumatic diseases (ARD) with high incidence of CVD [104]. Although targeted treatment has significantly decreased the disease-related mortality, life expectancy in ARD patients still remains low compared to the general population [105], mainly due to increased CVD [106–110]. There are several pathophysiologic processes that contribute to the development of CVD in patients with ARD, including myocardial/vascular inflammation [111–113], macro- and micro- vasculopathy [114, 115], and small epicardial, intramyocardial and/or subendocardial fibrosis due to inflammation and/or myocardial infarction [114, 116, 117]. These processes are not well evaluated by traditional imaging techniques, and an ideal diagnostic evaluation would include assessment of the acuity of heart involvement [113, 118, 119] and angiography of the great vessels with assessment of the arterial wall inflammatory process [116]. Iron overload states represent another relevant systemic disease that can affect the heart. Iron overload cardiomyopathy (IOC) results from genetic iron metabolism disorders (primary hemochromatosis) or multiple transfusions (thalassemia or myelodysplasia).

*Sex differences/prevalence:* ARD affect 8% of the population and approximately 78% of patients are women [120]. Sex differences are the result of various factors, including sex hormones, microchimerism, genes on X or Y chromosomes, X chromosome inactivation and differing responses to environmental factors. Estrogens may directly increase ARD in women by elevating auto-antibodies and amplifying autoreactive T- and B-cell responses [121]. Myocardial iron overload (MIO) is seen approximately 30% of patients with thalassemia [122, 123] Although men and women have a similar risk of iron accumulation, women have significant lower risk for cardiac dysfunction, heart failure and arrhythmias compared to men[122].

*Traditional evaluation:* The standard evaluation of ARD patients with suspected CVD often includes clinical history and examination, ECG, TTE, 24-h cardiac ECG monitoring, nuclear imaging and invasive x-ray coronary angiography, if necessary. However, the silent presentation of cardiac involvement in ARD patients is difficult to detect and may hamper comprehensive clinical evaluation and treatment. The limited tissue characterization of TTE leads to under-diagnosis of early myocardial involvement, which may lead to fatal arrhythmias despite preserved LV and RV function [124]. Nuclear imaging techniques may miss small perfusion/fibrosis defects due to inherently low spatial resolution. Annual ECG, 24 h ECG monitoring, and TTE are generally used in the evaluation and follow-up of patients with suspected MIO.

*Benefits for use of CMR* *(expert opinion):* CMR provides significant advantages over other imaging techniques in the assessment of CVD in ARD patients, owing to CMR’s high spatial resolution and ability to detect small myocardial and vascular changes. In patients with suspected IOC, the highly reproducible and sensitive CMR T2* technique has revolutionized management, providing noninvasive, quantitative and validated MIO assessment [118, 125, 126]. Initial cardiac T2* assessment should be performed as early as possible in the course of the disease. Annual CMR assessment is recommended, but can be repeated every 2 years in patients without MIO or in women given the lower CVD risk [126, 127]. Precise assessment of degree of iron deposition that can inform chelation therapy in patients with MIO. Comprehensive CMR examination allows for evaluation of the various pathophysiologic processes that may be present in patients with ARD and MIO. CMR adds to the diagnosis and management of CVD in ARD and MIO based on the assessment of myocardial ischemia and replacement or diffuse fibrosis [115, 117, 119, 128–130], disease acuity and extent [112, 114, 117, 128–132] due to either macro- or micro-vascular coronary artery disease. It also helps with etiology of silent/overt heart failure or rhythm disturbances [112, 133].High accuracy of stress CMR is valuable for early assessment of CAD [117].

### Suggested protocol

- bSSFP cine (short and long axis planes) for evaluation of cardiac structure and function
- Detection of myocardial edema/inflammation using a T2-based method (e.g. T2w imaging or T2-mapping) according to current expert recommendations [63]
- Detection of myocardial edema, hyperemia/capillary leak, necrosis and fibrosis using a T1-based method (e.g., early gadolinium enhancement, T1-mapping, ECV and/or T1w LGE) according to current expert recommendations [63]
- Stress-rest first pass perfusion for evaluation of myocardial ischemia (optional, if there is relevant clinical question)
- T2* mid short axis (optional basal, mid and apical short axis) when MIO is suspected

### CMR in female carriers of heritable cardiomyopathies

*Clinical scenario:* A genetic carrier is an individual with a mutation, typically recessive, in an allele or one copy of a gene [134]. Female carriers differ from males in recessive mutations on the X chromosome. Historically, female cardiogenetic carriers were thought to be immune to X-linked disease manifestations, having 2 X chromosomes. However, carriers may incur mild to severe phenotypic changes with mutations for dilated (such as Duchenne and Becher muscular dystrophy [135] and Danon’s disease [136] and hypertrophic (e.g. Anderson-Fabry disease) cardiomyopathies [137]. Disease penetrance often occurs later in life for carrier women vs. affected men. Cardioprotective therapies have been proven efficient when structural or functional abnormalities are present, even prior to symptom onset—a state known as ‘stage B cardiomyopathy’ [138]. Thus, clinical recognition of carrier status is important to facilitate cardiac risk assessment and management. With increasing acknowledgement of the risk of cardiomyopathy in female carriers, early detection with CMR provides guidance for deployment of effective therapies in female carriers of cardiomyopathy-causing mutations.

*Prevalence:* The prevalence of mutations that place women at risk of cardiomyopathy is not known but likely under-recognized, and genetic testing remains limited in its use to evaluate ‘idiopathic’ cardiomyopathy. Genetic testing of mothers or female siblings of males with X-linked cardiomyopathies are recommended and should be more widely practiced [68, 135–140].

*Traditional evaluation:* Female carriers may be screened by compiling a pedigree, clinical history, physical examination, ECG, and imaging, typically TTE. In the absence of cardiac symptoms, carriers may undergo no further testing. However, CMR is variably used to screen asymptomatic carriers or evaluate those with symptoms.

*Benefits for use of CMR in this scenario* *(expert opinion)*: CMR uniquely identifies myocardial changes resulting in female carriers well before typical measures of cardiac function are abnormal. Tissue characterization coupled with biventricular function quantification constitutes the major benefit of using CMR. Female mutation carriers of Duchenne, Danon and Anderson-Fabry’s diseases have varying degrees of myocardial damage by CMR even with normal LV size and function, before apparent heart failure or sudden death occur [136, 137, 141]

### Suggested protocol

- bSSFP cine imaging for cardiac functional and volumetric assessment according to societal guidelines [74]
- T1w LGE, typically showing epicardial or midwall fibrosis in X-linked cardiomyopathies, and to exclude ischemic damage [42, 140]
- Further tissue characterization using Tw imaging and/or parametric T1/T2 mapping, particularly T1 mapping in Anderson-Fabry disease carriers [142] or in unclear LV hypertrophy

## Safety considerations related to magnetic field and contrast media

i. Magnetic field considerations

CVD remains a major cause of morbidity and mortality in pregnant and post-partum women [143]. Although pregnancy is not a contraindication to magnetic resonance imaging (MRI), heating by radiofrequency pulses and effects of acoustic noise on the fetus have been raised as potential concerns. However, a retrospective study in 754 neonates who had 1.5 T MRI in utero showed no effect on hearing function or birth weight compared to control neonates [144]. Further in a large retrospective study in Canada that analyzed 1737 pregnancies with MRI, an exposure to MRI was not associated with a higher risk of stillbirth or neonatal death, congenital abnormalities, neoplasm or hearing loss when compared to 1.4 million pregnancies without MRI [145]. Therefore, recent results suggested that non-contrast enhanced MRI appears to be safe in pregnant women. Of note, due to increased heating effect and lack of safety data at 3 T, it is recommended to avoid MRI at > 1.5 T during pregnancy.

ii. Contrast media considerations

GBCA can cross the placenta and is excreted by the fetal kidneys into the amniotic fluid, and then returns to the fetal circulation by swallowing. Although the amounts of gadolinium chelate in the fetal tissues and amniotic fluid were much smaller than the maternal injected dose, prolonged recirculation of the contrast medium in fetal tissue can cause adverse effects [146]. In a retrospective study of Canadian provincial pregnancies from 2003 to 2015, GBCA MRI during pregnancy was associated with higher risk of stillbirth or neonatal death and a broad set of rheumatological, inflammatory, or infiltrative skin conditions, compared to the control group who did not undergo MRI during pregnancy [145]. Accordingly, GBCA should only be used if contrast-enhanced CMR is considered critical and the potential benefits justify the potential risk to the fetus [147]. When there is a very strong indication for contrast-enhanced CMR, the smallest possible dose of a macrocyclic GBCA may be given to the pregnant women [146].

Previously, some centers have recommended avoidance of breast feeding for 24 h after administrating GBCA in lactating women. However, less than 0.04% of the total maternal dose of intravenous GBCA passes into the breast milk over 24 h, with only a small fraction of this amount absorbed from the gastrointestinal tract [148].Therefore, according to the current guidelines [146, 147], breast feeding may be continued when macrocyclic GBCA are given to the mother.

## Conclusion

This document summarizes the recommendations of the SCMR Consensus Group on CMR Imaging for Female Patients with Cardiovascular Disease. After thorough review of current evidence and major society guidelines, and input from experts in the field, the group has proposed eight clinical applications where CMR is particularly useful in the assessment of female cardiovascular diseases.

The position statement does not consider CMR accessibility or availability of local expertise, but instead highlights the optimal utilization of CMR in women with cardiovascular disease. Finally, the ultimate goal of this position statement is to improve the health of female patients with cardiovascular disease by providing specific recommendations on use of CMR.

## Data Availability

Data sharing not applicable to this article as no datasets were generated or analysed during the current study.

## Abbreviations

2D: Two-dimensional
3D: Three-dimensional
ACC: American College of Cardiology
ACS: Acute coronary syndrome
AHA: American Heart Association
ARD: Autoimmune rheumatic diseases
BAV: Bicuspid aortic valve
BSA: Body surface area
bSSFP: Balanced steady state free precession
CAD: Coronary artery disease
CCTA: Coronary computed tomography angiography
CHD: Congenital heart disease
CMR: Cardiovascular magnetic resonance
CTA: Computed tomography angiography
CTRCD: Cancer therapy-related cardiac dysfunction
CVD: Cardiovascular disease
ECG: Electrocardiogram
ECV: Extracellular volume fraction
ESC: European Society of Cardiology
GBCA: Gadolinium based contrast agent
IHD: Ischemic heart disease
IOC: Iron overload cardiomyopathy
IVUS: Intravascular ultrasound
LAD: Left anterior descending coronary artery
LGE: Late gadolinium enhancement
LV: Left ventricle/left ventricular
LVEF: Left ventricular ejection fraction
LVOT: Left ventricular outflow tract
MI: Myocardial infarction
MIO: Myocardial iron overload
MINOCA: Myocardial infarction with non-obstructive coronary arteries
MRA: Magnetic resonance angiography
MRI: Magnetic resonance imaging
MUGA: Multi-acquisition gated angiography
MVO: Microvascular obstruction
NSTEMI: Non-ST-segment elevation myocardial infarction
NYHA: New York Heart Association
OCT: Optical coherence tomography
PET: Positron emission tomography
PPCM: Peripartum cardiomyopathy
RV: Right ventricle/right ventricular
RVEF: Right ventricular ejection fraction
SCMR: Society for Cardiovascular Magnetic Resonance
SPECT: Single-photon emission computerized tomography
STEMI: ST-segment elevation myocardial infarction
T1w: T1-weighted
T2w: T2 weighted
TTE: Transthoracic echocardiography
